# Tropomyosin3 overexpression and a potential link to epithelial-mesenchymal transition in human hepatocellular carcinoma

**DOI:** 10.1186/1471-2407-10-122

**Published:** 2010-04-01

**Authors:** Hye-Sun Choi, Seon-Hee Yim, Hai-Dong Xu, Seung-Hyun Jung, Seung-Hun Shin, Hae-Jin Hu, Chan-Kwon Jung, Jong Young Choi, Yeun-Jun Chung

**Affiliations:** 1 Department of Microbiology, School of Medicine, The Catholic University of Korea, 505 Banpo-dong, Socho-gu, Seoul 137-701, Korea; 2 Integrated Research Center for Genome Polymorphism, School of Medicine, The Catholic University of Korea, 505 Banpo-dong, Socho-gu, Seoul 137-701, Korea; 3 Department of Hospital Pathology, Seoul St Mary's Hospital, School of Medicine, The Catholic University of Korea, 505 Banpo-dong, Socho-gu, Seoul 137-701, Korea; 4 Department of Internal Medicine, Seoul St Mary's Hospital, School of Medicine, The Catholic University of Korea, 505 Banpo-dong, Socho-gu, Seoul 137-701, Korea

## Abstract

**Background:**

Since hepatocellular carcinoma (HCC) is one of the leading causes of cancer death worldwide, it is still important to understand hepatocarcinogenesis mechanisms and identify effective markers for tumor progression to improve prognosis. Amplification and overexpression of Tropomyosin3 (*TPM3*) are frequently observed in HCC, but its biological meanings have not been properly defined. In this study, we aimed to elucidate the roles of TPM3 and related molecular mechanisms.

**Methods:**

TPM3-siRNA was transfected into 2 HCC cell lines, HepG2 and SNU-475, which had shown overexpression of TPM3. Knockdown of TPM3 was verified by real-time qRT-PCR and western blotting targeting TPM3. Migration and invasion potentials were examined using transwell membrane assays. Cell growth capacity was examined by colony formation and soft agar assays.

**Results:**

Silencing TPM3 resulted in significant suppression of migration and invasion capacities in both HCC cell lines. To elucidate the mechanisms behind suppressed migration and invasiveness, we examined expression levels of Snail and E-cadherin known to be related to epithelial-mesenchymal transition (EMT) after TPM3 knockdown. In the TPM3 knockdown cells, E-cadherin expression was significantly upregulated and Snail downregulated compared with negative control. TPM3 knockdown also inhibited colony formation and anchorage independent growth of HCC cells.

**Conclusions:**

Based on our findings, we formulate a hypothesis that overexpression of TPM3 activates Snail mediated EMT, which will repress E-cadherin expression and that it confers migration or invasion potentials to HCC cells during hepatocarcinogenesis. To our knowledge, this is the first evidence that TPM3 gets involved in migration and invasion of HCCs by modifying EMT pathway.

## Background

Hepatocellular carcinoma (HCC) is one of the most common human malignancies and the third leading cause of cancer-related death in the world [1]. A number of studies have been suggesting the molecular mechanisms involved in hepatocarcinogenesis such as MAPK, EGFR, p53, Wnt, TGF-B, Ras and Rb pathways [2-5]. However, given that prognosis of the disease remains poor, it is still important to understand hepatocarcinogenesis mechanisms and to identify effective markers for early diagnosis and accurate prognostication which reflect biological phenomena well.

In our recent study which reported the chromosomal alterations in HCC by genome-wide array-CGH analysis, we found that a 1q21.3 locus was recurrently amplified and that a Tropomyosin 3 (*TPM3*) gene located in this region was coherently overexpressed in primary HCC [6]. This evidence suggests that overexpression of TPM3 may play a role in HCC tumorigenesis. TPM3 is an actin-binding protein present in skeletal and smooth muscle and some non-muscular tissues. In skeletal muscle, TPM3 mediates a myosin-actin response to calcium ions and takes part in the stabilization of cytoskeletal microfilaments [7]. On the contrary, the function of TPM3 in non-muscular tissues is still obscure.

Lines of evidence have suggested that non-muscular tropomyosins might be involved in tumor development. TPM3 was reported to be involved in hematopoietic tumorigenesis by forming a TPM3-ALK fusion through (1;2) translocation [8,9]. TPM3 is also known as an inducer of papillary thyroid carcinoma and chronic eosinophilic leukemia through a fusion with NTRK1 and PDGFRB [10,11]. In addition, tropomyosin family members have been reported to be related with tumor cell movement or invasion [12,13]. In Miyado et al.'s observation, the expression level of a low-molecular weight tropomyosin isoform, TM5/TM30nm, was higher in a highly metastatic mouse melanoma cell line than in a low-metastatic one [14]. This evidence suggests that overexpression of TPM3 may contribute to invasion or migration potentials of human malignancies including HCC, but molecular mechanisms behind this has not been explored.

In this study, we explored the biological roles of TPM3 in hepatocarcinogenesis and involved molecular mechanisms by TPM3 knockdown using small interfering RNA (siRNA) in human HCC cell lines.

## Methods

### HCC cell lines

HepG2 was obtained from ATCC (American Type Culture Collection, Manassas, VA) and maintained in DMEM (Gibco BLR, Gaithersburg, MD) supplemented with 10% FBS. SNU-739, 423, 449, 886, 475, 878, 387, 398, and 761 were obtained from the Korean cell-line bank (Seoul, Korea) and maintained in RPMI 1640 (Hyclone, Logan, UT) supplemented with 10% FBS at 37°C in humidified air containing 5% CO_2_. THLE-3 (a human normal liver cell) was purchased from ATCC (Manassas, VA) and maintained in DMEM supplemented with 10% FBS, 25 mM HEPES buffer and 100 U/ml of penicillin.

### siRNA oligonucleotides

We purchased two synthetic double-stranded oligonucleotides with the following sequences and introduced them into the pSilencer 3.1-H1 neo siRNA expression vector (Invitrogen, Carlsbad, CA); TPM3 RNAi-1, AGC AUU CUC CUU GUC UAA CUU CAG C: GCU GAA GUU AGA CAA GGA GAA UGC U; TPM3 RNAi-2, UAA CCU UCA UAC CUC UCU CAC UCU C: GAG AGU GAG AGA GGU AUG AAG GUU A. To verify sequence-specific effectiveness of TPM3-RNAi, we used a negative control siRNA (Invitrogen, Carlsbad, CA) that has no significant homology with any known sequences in the human genome.

### Transfection of TPM3 siRNAs

We adopted a forward transfection method. In brief, transfection was performed by adding the mixture of siRNA and the transfection reagent (lipofectamine RNiMAX, Invitrogen, Carlsbad, CA) onto the cells after the cell seeding. HepG2 and SNU475 cells were seeded at a density of 200,000 and 100,000 cells/well in six-well plates, respectively, and incubated for 24 hours at 37°C with 5% CO_2_. After 24 hour incubation, HCC cells were transfected with 100 nM siRNAs (2 TPM3 siRNAs and a negative control siRNA) using 1.25 μg/ml lipofectamine RNAiMax (Invitrogen, Carlsbad, CA) according to the manufacturer's instructions. After 48 hours following the transfection, HCC cells were harvested and silencing of the TPM3 expression was validated by real-time quantitative RT-PCR (qRT-PCR) and western blotting.

### Quantitative RT-PCR

Total RNA was extracted from the HCC cell lines using TRIzol (Invitrogen, Carlsbad, CA) according to the manufacturer's instructions. First-strand complementary DNA (cDNA) was synthesized from 5 μg of total RNA using oligo-dT primer and superscript II reverse transcriptase (Invitrogen, Carlsbad, CA). To determine the levels of *TPM3 *messenger RNA (mRNA) expression, real-time qRT-PCR was performed using Mx3000P QPCR System and software MxPro Version 3.00 (Stratagene, La Jolla, CA). Reaction mixture was composed of 1 × SYBR Green *Tbr *polymerase Master Mix (FINNZYMES, Finland), 0.5 × ROX and 20 pmol of each primer, and 10 ng of cDNA. Primers for *TPM3 *were 5'-GAGAGGTATGAAGGTTATTCA-3' for forward and 5'-ATCACCACCTTACGAGCCACC-3' for reverse. *GAPDH *was used as internal control. *GAPDH *primers were designed as 5'-GCGGGGCTCCAGAACATCAT-3' for forward and as 5'- CCAGCCCCAGCGTCAAGGTG-3' for reverse. RNA levels of *E-Cadherin *and *Snail *were measured using the following primers according to previous reports [15,16]; for Snail, 5' -AAGGATCTCCAGGCTCGAAAG-3' for forward and 5'-GCTTCGGATGTGCATCTTGA-3' for reverse; for E-cadherin, 5'-TCGACACCCGATTCAAAGTGG-3' for forward and 5'- TTCCAGAAACGGAGGCCTGAT -3' for reverse. The PCR program was as follows: denaturation at 95°C for 5 minutes; 40 cycles of 95°C for 30 seconds, 60°C for 30 seconds, and 72°C for 40 seconds followed by a 72°C elongation step for 6 minutes. Relative expression quantification was performed by the ΔΔCT method [17]. All the experiments were repeated three times and the mean value of intensity ratios with the SD was plotted for each case.

### Western blot analysis

Proteins were separated by 10% sodium dodecyl sulfate (SDS)-polyacrylamide gel electrophoresis (PAGE) and transferred onto polyvinylidene difluoride membranes (Millipore, Bedford, MA). The membrane was blocked with 5% non-fat dried milk in TBST(20 mM Tris-HCl, 150 mM NaCl, and 0.1% Tween 20, pH 7.5) for 2 hours and incubated overnight with antibodies against TPM3 (1:1,000 dilution; Abnova, Taipei, Taiwan), α-tubulin(1:1,000 dilution; Santa Cruz biotechnology, Santa Cruz, CA), Snail (1:500 dilution; Abcam, Cambridge, UK), E-cadherin (1:1000 dilution; Zymed, San Francisco, CA) at 4°C. After the wash with TBST buffer, membranes were incubated with horseradish peroxidase-conjugated anti mouse IgG secondary antibodies for 1 hour at room temperature and detected by enhanced chemiluminescence detection system (Amersham-Pharmacia Biotech, Braunschweig, Germany).

### Immunofluorescence staining

The samples of siTPM3 and siNEG transfected cells (SNU-475 and HepG2) were cytocentrifuged onto the slides and immediately fixed with ethyl alcohol for 30 minutes. For immunofluorescence staining, slides were exposed to 0.2% Tween 20 in PBS for 30 minutes and incubated overnight at 4°C with monoclonal antibodies against vimentin (1:100, clone V9, DakoCytomation, Glostrup, Denmark) and fibronectin (1:100, clone 568, Novocastra, Newcastle upon Tyne, UK). After thorough washing, cells were incubated with a 1:500 dilution of Alexa Fluor 488-conjugated goat anti-mouse IgG antibody (Invitrogen - Molecular Probes, Eugene, OR) for 30 minutes at room temperature in the dark. Staining of nuclei with diaminophenylindole (Molecular Probes) was also performed. Staining patterns were observed by a fluorescence microscope (Carl Zeiss, Axio Imager M1, Oberkochen, Germany) (400×).

### Migration and invasion assays

Migration of HCC cells was assayed using the transwell with 8-μm pore filters (Costar, Boston, MA). After filling the lower chamber with complete media, 2 × 10^4 ^HCC cells in 0.5 mL serum-free media were loaded onto the upper chamber. After incubation for 12 hours at 37°C, cells that migrated to the bottom surface of the membrane were fixed with methanol and stained with 0.5% crystal violet and then subjected to microscopic inspection. Cells on the top surface of the membrane were removed by wiping with a cotton swab. The numbers of cells were counted in five microscopic fields (×200). For the Matrigel invasion assay, the procedures were same as those for the cell migration assay, except that the transwell membrane was coated with 500 ng/μL of Matrigel (BD Biosciences, San Jose, CA) and incubated for 24 hours at 37°C.

### Colony formation and soft agar assays

For the colony formation assay, siRNA-treated and negative control-treated HCC cells (1 × 10^4^) were seeded in 10 cm dishes. Two weeks later, cells were washed with PBS buffer and stained with 0.5% crystal violet in 20% methanol for 20 minutes and the number of colonies was counted. For the soft agar assay, HCC cells were suspended in RPMI1640 containing 0.35% low melting agarose, and plated onto solidified 0.6% agarose containing RPMI1640 in six-well culture plates at a density of 1 × 10^5 ^cells per dish. The number and size of colonies were observed 3 weeks after seeding under the microscope (×40).

### Statistical Analysis

An independent samples t-test was used to test the significance of difference between groups and *P *values < 0.05 were considered significant. Data were analyzed using Stata version 10 software (Stata Corporation, College Station, TX).

## Results

### Elevated TPM3 expression in HCC cell lines

**Figure 1:**
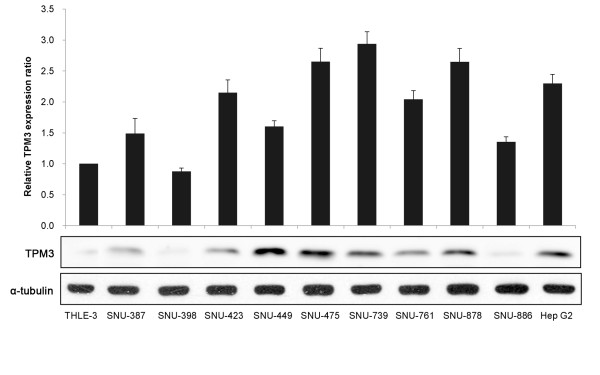
**TPM3 expression levels in various human HCC cell lines**. One normal human liver cell line (THLE-3) and 10 types of HCC cell lines were examined by TPM3-specific real-time qRT-PCR (top plot) and western blot (bottom plot). Human *GAPDH *gene was used as internal control for qRT-PCR and alpha-tubulin was used as internal control for western blot analysis. In the top plot, X axis represents cell lines and Y axis relative TPM3 expression ratios (tumor/normal). Error bars represent mean ± standard error of mean.

We firstly screened baseline TPM3 expression levels in 10 HCC cell lines. In eight out of the 10 HCC cell lines except for SNU-398 and SNU-886, both the mRNA (>1.5 fold) and protein expression levels of TPM3 were found to be increased with respect to the normal liver cell line (THLE-3) (Figure 1).

### Suppression of TPM3 expression by siRNA transfection

**Figure 2:**
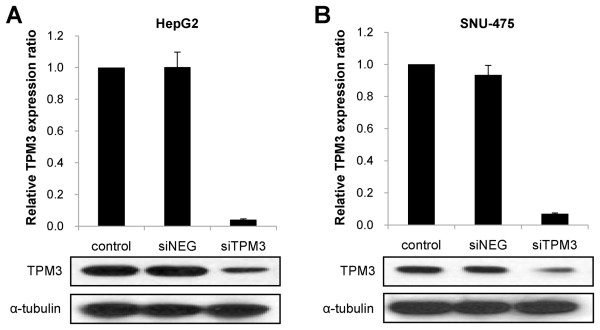
**Suppressed TPM3 expression after siTPM3 transfection into HepG2 (A) and SNU-475 (B)**. TPM3 expression was measured by real-time qRT-PCR (top plots of A and B). X axis represents samples and Y axis relative TPM3 expression ratio (siTPM or siNEG/control). Error bars represent mean ± standard error of mean. Human *GAPDH *gene was used as internal control. TPM3 expression was also measured by western blot (bottom plots of A and B). TPM3 band intensities of siTPM-treated cells are much weaker than those of siNEG-treated ones and control, while internal control bands are consistent. Alpha-tubulin was used as internal control for western blot analysis. siTPM3, siTPM3 transfected HCC cell line; siNEG, negative oligonucleotide (siNEG) transfected HCC cell line; control, HCC cell line without transfection.

For TPM3 knockdown, we transfected two siRNA constructs into those 8 cell lines which showed TPM3 overexpression compared with the THLE-3 on both mRNA and protein levels; siTPM3-1 and siTPM3-2 targeting exons 1 and 3/4, respectively. As a control, a negative oligonucleotide construct (siNEG) was transfected into the same 8 cell lines. As siTPM3-1 showed better knockdown effects between the two siRNA constructs and the best interfering efficiency was observed in SNU-475 and HepG2 cell lines (data not shown), all the downstream functional analyses were performed using siTPM3-1 (herein after called siTPM3) in these two cell lines. Figure 2 presents real-time qRT-PCR and western blotting results showing the repressed expression of TPM3 induced by transfecting siTPM3. In HepG2, relative TPM3 mRNA expression ratios (siTPM or siNEG/no transfection control) were 1.00 (95% CI 0.76-1.24) and 0.04(95% CI 0.03-0.06) in siNEG- and siTPM3-transfected cells, respectively (*P *< 0.0001). In SNU-475, relative TPM3 expression ratios were 0.91(95% CI 0.77-1.05) and 0.07(95% CI 0.06-0.08) in siNEG- and siTPM3-transfected cells, respectively (*P *< 0.0001). TPM3 protein expression in both siTPM-treated cells is much weaker than those in siNEG-treated ones, while alpha-tubulin bands are consistent (Figure 2A and 2B).

### Effects of TPM3 silencing on HCC cell migration and invasion

**Figure 3:**
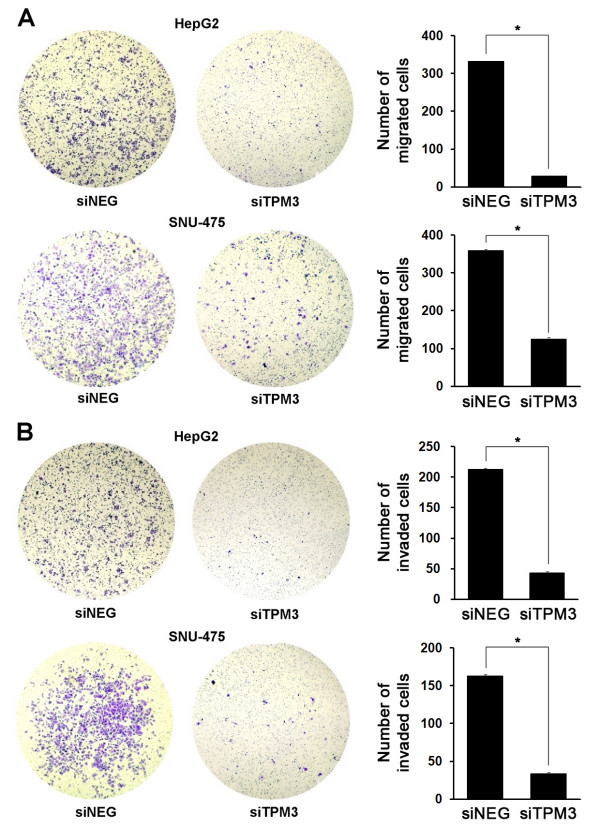
**Repressed migration and invasion in TPM3 knockdown HCC cell lines**. (A) Migration of siTPM and siNEG transfected HCC cell lines was examined using the Matrigel uncoated transwell membrane. After crystal violet staining, the numbers of colonies in five microscopic fields (X200) were counted. In HepG2, 332.0 in siNEG-treated cells (95% CI 323.0-341.0) *versus *29.3 in siTPM3-treated cells (95% CI 25.5-33.1), *P *< 0.0001; In SNU-475, 359.3(95% CI 350.6-368.1) *versus *125.3(95% CI 114.1-136.5), *P *< 0.0001. (B) Invasion of siTPM and siNEG transfected HCC cell lines was examined using the Matrigel coated transwell membrane. In HepG2, 212.7 in siNEG-treated cells (95% CI 206.4-218.9) *versus *43.0 in siTPM3-treated cells (95% CI 36.4-49.6), *P *< 0.0001; In SNU-475, 162.7(95% CI 156.4-168.9) *versus *33.7(28.5-38.8), *P *< 0.0001. Error bars represent mean ± standard error of mean. * represents *P *value < 0.05.

In order to explore the potential role of TPM3 on the invasiveness of HCC cells, we performed cell migration and invasion assays using siTPM3-treated HepG2 and SNU-475. Both migration and invasion capacities were found to be profoundly repressed in siTPM3-treated cells (Figure 3). In both cell lines, the numbers of migrated cells significantly decreased compared with siNEG control (*P *< 0.0001, Figure 3A). Repression of invasiveness by siTPM3 treatment was also observed. The numbers of the cells that passed through the Matrigel-coated membrane significantly decreased in siTPM3-treated cells compared with siNEG control (*P *< 0.0001, Figure 3B).

### E-cadherin and Snail expression in TPM3 knockdown HCC cells

**Figure 4:**
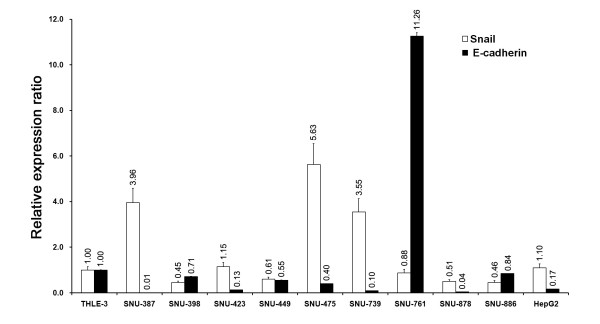
**Endogenous Snail and E-cadherin expression in 10 HCC cell lines and a normal liver cell line (THLE-3)**. Endogenous expression levels of Snail and E-cadherin were measured by real-time qRT-PCR before siTPM transfection. Total RNA extraction and qRT-PCR procedure were as described in Materials and Methods. Human *GAPDH *gene was used as internal control for qRT-PCR. X axis represents cell lines and Y axis represents relative expression ratios of each gene (Cell lines/THLE-3). Error bars represent mean ± standard error of mean. Open bar represents Snail expression and closed bar represents E-cadherin expression.

**Figure 5:**
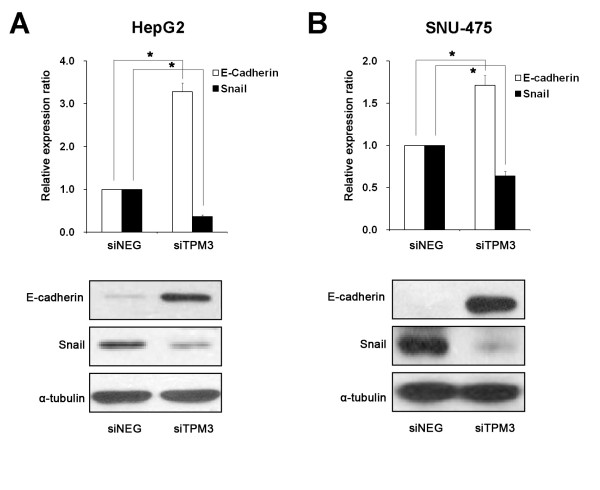
**Changes in E-cadherin and Snail expression after TPM3 knockdown in HepG2 (A) and SNU-475(B)**. After TPM3 knockdown, E-cadherin and Snail expression levels were measured by real-time qRT-PCR (top plots). * represents *P *value < 0.05. X axis represents samples and Y axis represents relative expression ratios of E-cadherin and Snail (siTPM/siNEG). Human *GAPDH *gene was used as internal control for qRT-PCRs. E-cadherin and Snail expression levels were also measured by western blot (bottom plots). Alpha-tubulin was used as internal control for western blot analysis. siTPM3, siTPM3 transfected HCC cell line; siNEG, negative oligonucleotide transfected HCC cell line. Error bars represent mean ± standard error of mean. * represents *P *value < 0.05.

To explore the potential mechanisms of reduced migration and invasion in TPM3 knockdown HCC cells, we examined the expression patterns of E-cadherin and Snail, a known factor to repress E-cadherin expression by binding to E-boxes of the E-cadherin promoter in cancers. Before knockdown, we measured the endogenous levels of E-cadherin and Snail in the 10 HCC cell lines and THLE-3 as a reference (Figure 4). Of the eight cell lines showing relative TPM3 overexpression with respect to THLE-3, five cell lines (SNU-387, 423, 475, 739, and HepG2) showed upregulated Snail and downregulated E-cadherin levels. Two of the eight cell lines with TPM3 overexpression (SNU-449 and 878) also showed the Snail up- and E-cadherin downregulated pattern, but the endogenous Snail levels were lower than that in THLE-3. In case of SNU-761, although it showed TPM3 overexpression, the expression pattern of Snail and E-cadherin was opposite to those of other 7 cell lines. The 2 cell lines without TPM3 overexpression (SNU-398 and 886) did not show Snail upregulation. When we knocked down TPM3 in HepG2 and SNU-475, the expression levels of Snail and E-cadherin became reversed in both cell lines; Snail expression was significantly decreased and E-cadherin was significantly increased compared with the siNEG transfection control on both mRNA and protein levels (Figure 5A and 5B).

### Vimentin and fibronectin expression in TPM3 knockdown HCC cells

**Figure 6:**
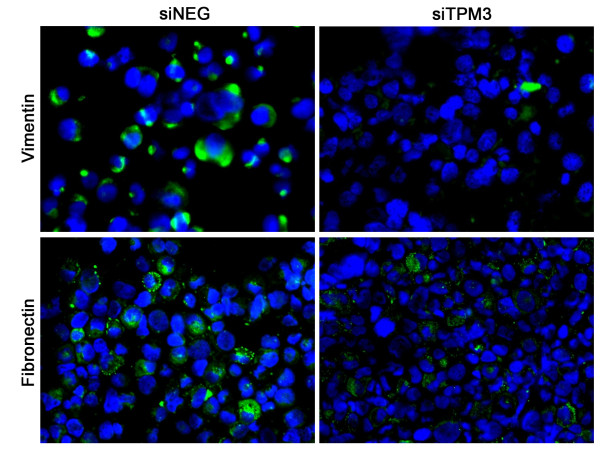
**Expression profiles of vimentin and fibronectin after TPM3 knockdown in SNU-475**. Vimentin (upper boxes) and fibronectin (lower boxes) expression patterns were compared between siTPM3- and siNEG-transfected SNU-475 by immunofluorescence staining (green for vimentin and fibronectin stain; blue for nuclear DAPI stain). siTPM3, siTPM3 transfected SNU-475; siNEG, siNEG transfected SNU-475. ×400

To further verify the changes of EMT-related phenotypes in TPM3 knockdown cells, we examined the expression of vimentin and fibronectin in cells with and without siTPM3 transfection. In SNU-475, endogenous vimentin and fibronectin were strongly expressed in the cytoplasm of control cells, but both signals decreased in siTPM3-treated cells (Figure 6). Especially, the decrease of vimentin expression after TPM3 knockdown was more noticeable than that of fibronectin. The profile in HepG2 was similar to that in SNU-475, but less prominent due to initially weaker vimentin and fibronectin signals than those in SNU-475 (data not shown).

### Inhibited tumor cell growth after TPM3 silencing

**Figure 7:**
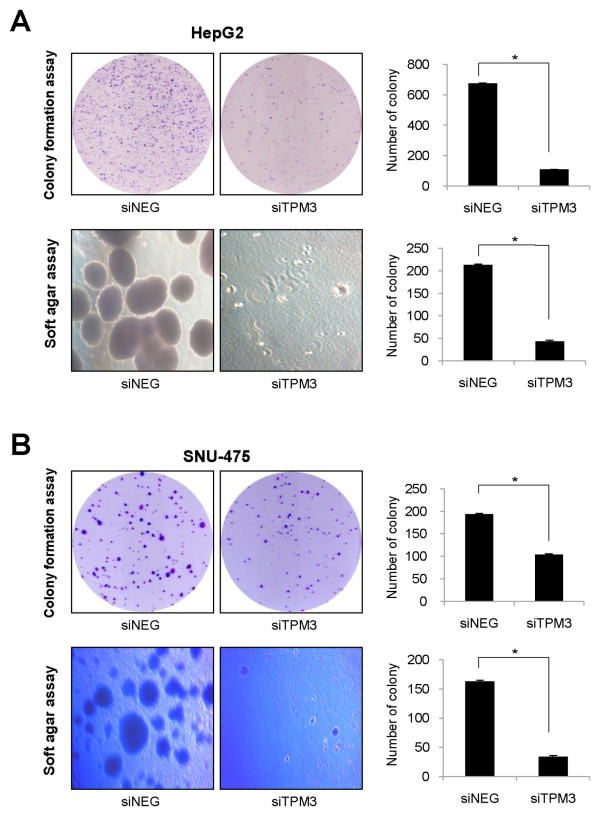
**Inhibited tumor cell growth in TPM3 knockdown HepG2 (A) and SNU-475 (B)**. Top plots of A and B are colony formation assay results. Bottom plots are soft agar assay results. In both the colony formation and anchorage independent growth assays, the number of colonies were counted in siTPM and siNEG plates. siTPM3, siTMP3 transfected HCC cell line; siNEG, negative oligonucleotide (siNEG) transfected HCC cell line. * represents *P *value < 0.05.

In addition to the effects on tumor cell migration and invasion, we also assessed the effect of TPM3 knockdown on HCC cell growth (Figure 7). First, we performed the colony formation assay. The numbers of colonies in siTPM3 treated cells were significantly reduced compared with those in siNEG treated cells; In HepG2, 685.7(95% CI 663.1-708.2) in siNEG-treated cells *versus *108.3(95% CI 104.5-112.1) in siTPM3-treated cells, *P *< 0.0001; In SNU-475, 190.0(95% CI 176.2-203.8) *versus *107.3(95% CI 92.8-121.9), *P *< 0.0001 (Figure 7A and 7B). We next examined the TPM3 knockdown effect on the anchorage independent growth of HCC cells by the soft agar assay. The numbers and sizes of anchorage-independent colonies were significantly lower in siTPM3-treated cells than those in siNEG-treated ones; In HepG2, 212.7 (95% CI 206.4-218.9) in siNEG-treated cells *versus *43.0(95% CI 36.4-49.6) in siTPM3-treated cells, *P *< 0.0001; In SNU-475, 162.7(95% CI 156.4-168.9) *versus *33.7(95% CI 28.5-38.8), *P *< 0.0001 (Figure 7A and 7B).

## Discussion

Previously, we reported a recurrent chromosomal amplification on the 1q21.3 region and related overexpression of the *TPM3 *gene and suggested its oncogenic potential in hepatocarcinognenesis [6]. Subsequently, we conducted this study to elucidate the biological effects of TPM3 overexpression in hepatocarcinogenesis using the RNA interference (RNAi) technology to knockdown the expression of TPM3. We found that TPM3 knockdown profoundly repressed the migration and invasion potentials of HCC cells compared with the same cell lines without siTPM3 treatment. These findings are accordant with the previous reports which suggested that expression of a tropomyosin isoform was higher in highly metastatic mouse tumor cells than in the cells with lower metastatic rate [14].

To explore the mechanisms behind reduced migration and invasion in TPM3 knockdown HCC cells, we examined whether transfection of siTMP3 would affect the levels of E-cadherin expression in HCC cells. Downregulation of E-cadherin expression is one of the well-known hallmarks of tumor metastasis in HCC and an indicator of EMT onset [18-21]. In our study, E-cadherin expression was found to be reversed from low to high through siTPM3 treatment. In previous HCC studies, repressed expression or mutation of E-cadherin was correlated with a histological grade, vascular invasion and intrahepatic metastasis through losing cell adhesion and increasing cell mobility [16,22,23]. We further examined the expression of Snail in TPM3 knockdown HCC cells showing upregulated E-cadherin, because the Snail transcription factor has been known to repress E-cadherin expression by binding to E-boxes in the E-cadherin promoter in cancers including HCC [17,24,25]. Snail is also known as a key regulatory molecule inducing EMT [17,22,26]. We found that Snail expression was significantly more repressed in siTPM3-treated HCC cell lines than in the untreated cell lines. Although directions of the effect were opposite, there have been studies which reported that overexpression of Snail increases the invasiveness of HCC [17,27,28]. Based on our observations and previous reports, it can be hypothesized that overexpression of TPM3 in the cytoplasm may activate Snail which will subsequently repress E-cadherin expression in the nucleus and that this event can confer migration or invasion potentials to cancer cells during hepatocarcinogenesis. Snail activation by TPM3 could be achieved through the direct interaction or activation of TPM3 downstream signaling pathways. However, we could not find the significant positive correlation between endogenous TPM3 and Snail mRNA levels, partly due to the limited number of cell lines we studied.

To see phenotypic consequences of TPM3 knockdown in HCC cells, we examined vimentin and fibronectin expression. Vimentin and fibronectin are the mesenchymal cell markers associated with EMT and known to be upregulated in migratory cells [29]. In siTPM3 transfected cells, the expression levels of vimentin and fibronectin decreased compared with the untreated cells. All these results support that TPM3 overexpression could affect migration or invasion potentials through activating EMT.

We also observed that TPM3 knockdown lowered colony formation and anchorage independent growth. In previous observations, upregulation of Snail was found to be associated with tumor cell survival and aggressive behavior of cancer [30,31]. Taken together, it could be suggested that reduced tumor cell growth in siTPM3 treated HCC cells might be due to TPM3 knockdown-related downregulation of Snail. In addition, in our unpublished study, the combined use of TPM3 knockdown and chemotherapeutic agents have been more effective to reduce tumor cell viabilities than the use of chemotherapeutic agents only. This is also coherent with the previous reports suggesting that Snail expression is critical for cancer cells to acquire chemoresistence [30,31].

There are several limitations in this study. First, we did not examine the direct binding of TPM3 to Snail. TPM3-Snail co-immunoprecipitation or other experimental verification of the correlation of these two molecules would be necessary. Second, although repression of colony formation and anchorage independent growth was observed in TPM3 knockdown cells, it cannot automatically prove the biological consequences of TPM3 overexpression in hepatocarcinogenesis. Larger-scale screening of TPM3 expression profile in invasive primary HCCs and TPM3 overexpression experiment in normal liver cell lines will provide more direct evidence to support its oncogenic potential. Lastly, it is unclear whether the TPM3-Snail pathway is hepatocarcinogenesis-specific or not. Examining other types of cancers will be required to clarify this possibility.

## Conclusion

In this study, we demonstrated that TPM3 knockdown profoundly repressed migration and invasion of HCC cell lines. Based on our findings, we formulate a hypothesis that overexpression of TPM3 activates Snail expression, which will repress E-cadherin expression and confer migration or invasion potentials to HCC cells during hepatocarcinogenesis. To our knowledge, this is the first evidence that TPM3 gets involved in migration and invasion of HCC by activating Snail mediated EMT pathway. This study will help to understand invasion or metastasis mechanisms in HCC and to define therapeutic targets inhibiting them.

## List of Abbreviations

(TPM3): tropomyosin3; (HCC): hepatocellular carcinoma; (EMT): epithelial-mesenchymal transition; (siRNA): small interfering RNA; (RNAi): RNA interference.

## Competing interests

The authors declare that they have no competing interests.

## Authors' contributions

HSC was involved in the design of this study and execution of most experiments and drafted the manuscript. SHY and HJH participated in the design of this study, statistical analysis, and writing the manuscript. CKJ performed immunofluorescence staining and western blot analysis, HDX, SHJ and SHS assisted experimental procedures such as migration/invasion and cytotoxicity assays. JYC participated in the design of this study and partly contributed to funding. YJC proposed this study, organized the research team, interpreted all the data, and participated in writing the manuscript.

## Pre-publication history

The pre-publication history for this paper can be accessed here:

http://www.biomedcentral.com/1471-2407/10/122/prepub
